# Men and women differ in the neural basis of handwriting

**DOI:** 10.1002/hbm.24968

**Published:** 2020-02-24

**Authors:** Yang Yang, Fred Tam, Simon J. Graham, Guochen Sun, Junjun Li, Chanyuan Gu, Ran Tao, Nizhuan Wang, Hong‐Yan Bi, Zhentao Zuo

**Affiliations:** ^1^ Key Laboratory of Behavioral Science, Center for Brain Science and Learning Difficulties, Institute of Psychology Chinese Academy of Sciences Beijing China; ^2^ Department of Psychology University of Chinese Academy of Sciences Beijing China; ^3^ Center for Language and Brain Shenzhen Institute of Neuroscience Shenzhen China; ^4^ Physical Sciences Platform Sunnybrook Research Institute Toronto Canada; ^5^ Department of Medical Biophysics University of Toronto Toronto Canada; ^6^ Department of Neurosurgery First Medical Center of Chinese PLA General Hospital Tianjin China; ^7^ Department of Chinese and Bilingual Studies The Hong Kong Polytechnic University Kowloon Hong Kong China; ^8^ Artificial Intelligence and Neuro‐informatics Engineering (ARINE) Laboratory, School of Computer Engineering Jiangsu Ocean University Lianyungang China; ^9^ State Key Laboratory of Brain and Cognitive Science Institute of Biophysics, Chinese Academy of Sciences Beijing China; ^10^ The Innovation Center of Excellence on Brain Science Chinese Academy of Sciences Beijing China; ^11^ Sino‐Danish College University of Chinese Academy of Sciences Beijing China

**Keywords:** Exner's area, fMRI, handwriting, sex differences

## Abstract

There is an ongoing debate about whether, and to what extent, males differ from females in their language skills. In the case of handwriting, a composite language skill involving language and motor processes, behavioral observations consistently show robust sex differences but the mechanisms underlying the effect are unclear. Using functional magnetic resonance imaging (fMRI) in a copying task, the present study examined the neural basis of sex differences in handwriting in 53 healthy adults (ages 19–28, 27 males). Compared to females, males showed increased activation in the left posterior middle frontal gyrus (Exner's area), a region thought to support the conversion between orthographic and graphomotor codes. Functional connectivity between Exner's area and the right cerebellum was greater in males than in females. Furthermore, sex differences in brain activity related to handwriting were independent of language material. This study identifies a novel neural signature of sex differences in a hallmark of human behavior, and highlights the importance of considering sex as a factor in scientific research and clinical applications involving handwriting.

## Graphical abstract

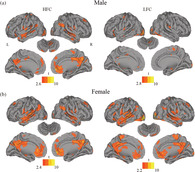

## 1 INTRODUCTION

It is still controversial whether, and to what extent, sex differences exist in language in the general population. Some studies have demonstrated that females outperform males in language comprehension (Chiu & McBride‐Chang, 2006; Logan & Johnston, 2010; Stoet & Geary, 2013) and language production (Leaper & Smith, 2004). However, meta‐analyses have shown that sex differences in linguistic variables are small or trivial (effect size *d* < 0.2) (Janet S Hyde & Linn, 1988; Zell, Krizan, & Teeter, 2015), favoring the sex similarities hypothesis (Janet Shibley Hyde, 2005). At the neural level, cerebral lateralization has been advocated as a key origin of sex differences in language (B. A. Shaywitz et al., 1995). For example, previous neuroimaging studies have found that during language tasks, females showed more bilateral activation in language‐related regions than males, including the inferior frontal gyrus, posterior superior temporal gyrus, and fusiform gyrus (Baxter et al., 2003; Burman, Minas, Bolger, & Booth, 2013; S. E. Shaywitz et al., 1998). This was found in neuroimaging studies of children (Bitan, Lifshitz, Breznitz, & Booth, 2010; Burman, Bitan, & Booth, 2008) as well as adults (Clements et al., 2006; B. A. Shaywitz et al., 1995). Using network analysis of functional magnetic resonance imaging (fMRI) data, a recent study demonstrated marked sex differences in functional integration and segregation among brain regions related to semantic processing (Xu, Liang, Ou, Li, Luo, & Tan, 2019). However, meta‐analyses with large sample sizes and stringent statistical comparisons of lateralization indices have failed to detect robust differences in cerebral lateralization during language processing (Hirnstein, Westerhausen, Korsnes, & Hugdahl, 2013; Ihnen, Church, Petersen, & Schlaggar, 2009; Sommer, Aleman, Bouma, & Kahn, 2004).

Handwriting is also an essential language skill that has been understudied in the context of sex differences. Beyond its communication function, handwriting is vital to reading acquisition and development (Graham & Hebert, 2011; James & Atwood, 2009; Nakamura et al., 2012; Tan, Spinks, Eden, Perfetti, & Siok, 2005). Interestingly, unlike other language skills, behavioral observations have shown reliable sex differences in handwriting (Camarata & Woodcock, 2006; Nowell & Hedges, 1998; Reilly, Neumann, & Andrews, 2019; Reynolds, Scheiber, Hajovsky, Schwartz, & Kaufman, 2015). Specifically, blind raters have successfully distinguished writing by males and females, with a large effect size (*d* = 0.75) (Beech & Mackintosh, 2005). There is also ample evidence from studies in large cohorts showing a female advantage in various handwriting tasks, including written composing and written fluency (Nowell & Hedges, 1998; Reilly et al., 2019; Reynolds et al., 2015; Scheiber, Reynolds, Hajovsky, & Kaufman, 2015). These differences were stable across historical time (Reilly et al., 2019) and the lifespan (Camarata & Woodcock, 2006).

Why does a salient sex difference occur in handwriting? Conceptually, handwriting involves multiple cognitive and motor operations, which can be broadly divided into two components: the “central” and “peripheral” processes. The former process refers to a linguistic component, involving the retrieval of appropriate words and correct orthographic forms via orthographic long‐term memory or phoneme‐to‐grapheme conversion. The latter process supports motor processing, involving the retrieval of motor forms via allographic/letter‐shape conversion and guidance of specific motor programs (Ellis, 1982). Multiple brain regions have been associated with handwriting, including the frontal motor cortex, the superior parietal lobule, the inferior temporal gyrus (fusiform gyrus) and the cerebellum (Planton, Jucla, Roux, & Démonet, 2013; Purcell, Turkeltaub, Eden, & Rapp, 2011). In particular, the posterior middle frontal gyrus (Exner's area) is a region that is proposed to support the link between orthographic and motoric programs (Planton et al., 2013; F. E. Roux et al., 2009). The intraparietal lobule/superior parietal lobule and the cerebellum are believed to support sensorimotor control associated with handwriting (Harrington, Farias, Davis, & Buonocore, 2007; Planton et al., 2013; Planton, Longcamp, Péran, Démonet, & Jucla, 2017). The left postcentral gyrus has consistently been found to be involved in handwriting (Katanoda, Yoshikawa, & Sugishita, 2001; Planton et al., 2013; Purcell et al., 2011), although the specific role of this region remains unclear. Finally, the left inferior temporal cortex including the mid‐fusiform gyrus is thought to support orthographic retrieval in handwriting (Nakamura et al., 2000; Planton et al., 2013; J. Purcell et al., 2011). In addition to language, handwriting is also a special form of fine motor. Generally, females usually perform better than males in fine motor skill (Hall & Kimura, 1995; Halpern, 1997), and males and females have been found to differ in utilizing cortical and subcortical regions to carry out motor control (Lissek et al., 2007; Rubia et al., 2013). Accordingly, language and motor networks, and their interactions, are candidates for the basis of sex differences in handwriting. To date, however, no studies have been undertaken that report sex differences in brain organization of handwriting.

To fill this gap in knowledge, the present study was carefully designed to examine sex differences in adults while they performed handwriting tasks during fMRI. Writing skill is mature and stable in adults, thus controlling for developmental and maturational factors that have been found to influence the presence of sex differences significantly (Hirnstein et al., 2013; Scheiber et al., 2015). Moreover, to measure writing‐specific sex differences appropriately, other handwriting‐related variables including reading, cognitive, and basic visual‐motor skills were matched between males and females. Word frequency is a vital language factor that influences orthographic access and motor execution during handwriting (Kandel & Perret, 2015; Qu, Zhang, & Damian, 2016; S. Roux, Mckeeff, Grosjacques, Afonso, & Kandel, 2013; Zhang & Cheng, 2014). Previous fMRI studies have demonstrated that word frequency modulates brain activity related to handwriting in superior/middle frontal gyrus, inferior parietal lobule and fusiform gyrus (Rapp & Dufor, 2011; Yang et al., 2018). Thus, word frequency was taken into account to test the influence of this language factor on sex differences in handwriting.

Hypothesis testing was conducted with the expectation of group differences in the mean brain activity of (a) Exner's area (Planton et al., 2017; F. E. Roux et al., 2009); (b) regions for orthographic processing (i.e., fusiform gyrus) that have been reported to show sex differences in a reading task (Chen et al., 2016) and an orthographic judgment task (Burman et al., 2013); and (c) the superior/intraparietal lobule and cerebellum (Planton et al., 2013; Purcell et al., 2011), given the likelihood of sex differences at the final stage of motor execution in handwriting behavior.

## 2 METHODS

### 2.1 Participants

Fifty‐three adults were recruited to participate in the study (27 males, mean age = 22.78 years; 26 females, mean age = 21.81 years). All participants were native Chinese speakers and were right‐handed as assessed by a handedness inventory (Snyder & Harris, 1993), while sex was self‐reported. The participants were physically healthy and reported no history of neurological disease or psychiatric disorder. The study was approved by the ethics committee of the Institute of Psychology, Chinese Academy of Sciences, and the methods were carried out in accordance with the approved guidelines. Free and informed consent was obtained from each participant prior to the experiment.

A series of reading, cognitive and visual‐motor tests were conducted to match the levels of possible confounds between males and females. Reading is very closely related to writing (Tan et al., 2005), and thus, comprehensive reading‐related tests were undertaken, including reading accuracy, reading fluency and orthographic skill. Reading accuracy was evaluated using a Chinese character reading test, in which 179 single Chinese characters were presented and participants were asked to compose meaningful words or phrases using the target characters. Each correct response scored one point, and the final score was calculated based on the norm from 100 college students. Reading fluency was evaluated by reading aloud 40 two‐characters words as quickly and accurately as possible, and the completion time was used as the score. In addition, a radical search task was employed to examine orthographic skill, in which participants were required to mark characters with a specific radical (“木,” mu4, wood) from 180 randomly arranged characters that might or might not include the designated radical (Siok & Fletcher, 2001). One correct mark scored 1 point.

Cognitive testing consisted of sustained attention and verbal working memory tests. Sustained attention was assessed by using a cancelation test, in which participants were instructed to search and mark the target number (“3”) as quickly and accurately as possible within 3 min. The score were calculated by this equation: score = attack −(false alarms+0.5*omission). A digit span task was employed for evaluating verbal working memory in which participants were asked to repeat a series of digits in forward (range 3 to 12 digits) and backward order (range 3 to 10 digits). The test was terminated when participants failed in two consecutive trials of the same length, and the working memory span was the number of digits in the last successful trials.

Finally, a visual‐motor integration test was conducted to assess low‐level visual‐motor skill in which participants were asked to copy 12 geometric symbols varying in visual complexity as accurately as possible. Two independent evaluators assessed the similarity between samples and participants' responses using a 7‐point scale, and the score was the sum of each symbol. The inter‐rater reliability of the assessment was good (intra‐class correlation coefficients [ICC] = 0.86).

There were no differences in all reading, cognitive and motor tests between males and females, suggesting that these abilities were well‐matched between groups. Detailed information of all the participants was presented in Table 1.

**Table 1:** Demographic characteristics of participants and behavioral results

	Males (*N* = 27) mean (*SD*)	Females (*N* = 26) mean (*SD*)	*p* values
Age (in years)	22.78 (2.35)	21.81 (2.04)	.089
Handedness	All right‐handed	All right‐handed	
Education (in years)	15.11 (1.57)	14.38 (1.52)	.095
Reading			
Accuracy	1,703 (350)	1,764 (367)	.534
Fluency (in s)	17.19 (2.7)	17.68 (3.33)	.555
Orthography	80.26 (10.36)	82.5 (10.34)	.435
Cognitive skills			
Sustained attention	44.03 (3.92)	45.32 (2.6)	.166
Working memory (forward)	9.41 (1.15)	9.35 (1.09)	.844
Working memory (backward)	7.3 (1.92)	7.35 (2.33)	.843
Visual‐motor integration	48.31(6.56)	48.56(8.49)	.907

Abbreviations: *SD*, standard deviation; s, second.

### 2.2 Stimuli and task procedure

Participants were instructed to perform a handwriting task (copying Chinese characters) and a control task (drawing nonsense symbols). Thirty characters were selected for presentation, of which one half were of “high frequency” (1,500 times per million) and the other half were of “low frequency” (< 5 times per million), according to the *Modern Chinese Frequency Dictionary* (1986). Visual complexity measured by the mean number of strokes was matched between high‐frequency and low‐frequency characters. To control for activation elicited by motor and basic visual processing, participants also performed a control task in which they were instructed to copy nonsense symbols. Participants were instructed to write characters and draw symbols with matched duration and size, while minimizing movements of their upper arm and forearm (thus minimizing potential head motion artifacts in the fMRI data).

Self‐paced (natural) writing is a commonly used task paradigm in investigations of the neural basis of handwriting (Berninger, Richards, & Abbott, 2015; Longcamp et al., 2014; Planton et al., 2017). Several advantages of a natural task in examining neural correlates of motor processing have been proposed (Maccotta, Zacks, & Buckner, 2001; Moritz, Carew, McMillan, & Meyerand, 2005). First, self‐paced handwriting might be close to real experience in daily life, which is beneficial for exploring the inherent sex differences in handwriting. Second, some confounding factors, such as attentional drift and task difficulty, could be minimized in the self‐paced task (Diciotti et al., 2007). This advantage is applicable to the examination of sex differences in handwriting, as behavioral studies consistently showed that males perform worse than females in handwriting (Reilly et al., 2019; Reynolds et al., 2015; Scheiber et al., 2015). Thus, in the present study, the brain mechanisms of sex differences were investigated in natural writing status, in which participants were instructed to write or draw at the speed that they use in daily life.

A block design was employed, consisting of six blocks of copying characters (three blocks for high‐ and low‐frequency characters, respectively) and three blocks of drawing symbols, in pseudo‐random order. Each block included visual presentation of instructions for 2 s followed by five trials. In each trial, a “+” symbol was first presented visually and centrally for 0.3 s, followed by presentation of a character stimulus for 1 s and then a response period of 4.7 s. Three blocks of central fixation each with 12 s duration were also interspersed among the task and control blocks as a “rest” condition. The total duration was 318 s. Detailed information about the experimental design has been reported previously (Yang et al., 2018).

Handwriting data were recorded using a tablet system specially developed for use in fMRI experiments. The tablet system includes a touch‐sensitive surface, a force‐sensitive stylus and an adjustable support frame, and is MRI‐safe without significantly degrading fMRI data quality (Karimpoor et al., 2018; Tam, Churchill, Strother, & Graham, 2011). The support frame was adjusted carefully for each participant to ensure that handwriting and drawing could be undertaken comfortably throughout the imaging session, and to enable tablet interaction with the forearm or wrist resting on the support such that there was no fatigue from handwriting against gravity.

### 2.3 Imaging acquisition

Imaging was performed using a 3 T MRI system (MAGNETOM Prisma^fit^, Siemens, Erlangen, Germany) at the Beijing MRI Center for Brain Research of the Chinese Academy of Sciences. Functional MRI time series data with blood oxygenation level‐dependent (BOLD) contrast were acquired using a two‐dimensional, T2*‐weighted, gradient‐echo echo planar imaging (EPI) sequence (Moeller et al., 2010) (repetition time TR = 1,000 ms, echo time TE = 30 ms, slices thickness = 2.2 mm, in‐plane resolution = 2.2 mm × 2.2 mm, flip angle θ = 45°, 64 axial slices). High spatial resolution anatomical images were acquired using a three‐dimensional T1‐weighted, magnetization‐prepared rapid acquisition gradient echo (MPRAGE) sequence (TR = 2,200 ms, TE = 2.08 ms, slice thickness = 1 mm, TI =1,000 ms, in‐plane resolution = 1.0 mm × 1.0 mm and θ = 8°).

### 2.4 Post‐imaging pen‐and‐paper writing tests

After fMRI, pen‐and‐paper writing tests were administered to all participants that required them to copy 40 Chinese characters using the “natural” writing style. Half of the characters were high‐frequency characters, and half were low‐frequency characters. To avoid practice effects, the characters used in the post‐imaging measure were different from those used in the fMRI task, but the visual complexity and frequency of characters were matched.

## 3 DATA ANALYSIS

### 3.1 Behavioral data

#### 3.1.1 fMRI task performance

Writing latency and writing duration were analyzed separately. The former was defined from the onset of response stimuli to the beginning of writing/drawing, and the latter was defined from the start of the response (first contact with the tablet) to the end of the last written or drawn stroke of the response. A 2 (sex: male vs. female) by 3 (stimulus type: high‐frequency character vs. low‐frequency character vs. symbols) analysis of variance (ANOVA) was conducted.

#### 3.1.2 Post‐imaging pen‐and‐paper writing performance

Writing quality was evaluated by two independent (one male) examiners using a 7‐point scale (1 = very bad and 7 = very good) based on the natural written scripts. The inter‐rater reliability was high (ICC = 0.91). This assessment was based on six dimensions, including stroke form, slant, organization of radicals, neatness, average size and overall appearance (Gimenez et al., 2014). The score were the sum of each dimension's score. Writing speed was the average completion time of copying.

In addition, following the method of previous studies (Beech & Mackintosh, 2005; Hamid & Loewenthal, 1996), a sex rating measure was adopted for the written scripts. Another 45 participants (20 males, mean age = 22.98 years) were recruited to make sex judgments, including a dichotomous judgment (male/female) and a 7‐point scale judgment (1 = very masculine; 7 = very feminine). The raters were recruited from the same source population as the writers, ensuring that they have similar experiences of handwriting, and are thus capable of detecting the cues from the features of written scripts on sex judgments. Independent two‐sample *t* tests were applied to investigate sex differences in writing quality, writing test completion time, and sex rating scores.

### 3.2 fMRI data

#### 3.2.1 Preprocessing

Image preprocessing and statistical analyses were conducted using SPM8 freeware (http://www.fil.ion.ucl.ac.uk/spm/, Wellcome Department of Cognitive Neurology, University College London, London). The fMRI time series data for each participant were first corrected for head motion, and the corrected images were co‐registered to the associated anatomical imaging data. The anatomical images were transformed into Montreal Neurological Institute (MNI) stereotactic space, and the resulting transformation parameters were then applied to yield fMRI time series data normalized in MNI space with cubic voxels at 2 mm × 2 mm × 2 mm spatial resolution. These images were then spatially smoothed using an isotropic Gaussian kernel template with 6 mm full‐width at half‐maximum. The data for one male participant were not examined further, because the exclusion criteria for head motion were exceeded (>2.5 mm translation or > 2.5° rotation). Data for another female participant were excluded due to substantial signal loss in the prefrontal cortex.

#### 3.2.2 Whole‐brain activation analysis

The general linear model (GLM) method was used to generate activation maps for handwriting characters and drawing symbols for each participant. The GLM design matrix included the block design time series convolved with a canonical hemodynamic response function. To minimize residual motion artifacts, head movement parameters (estimated with six degrees of freedom during the motion correction step) were included in the design matrix as nuisance covariates. The data were high‐pass filtered at 0.008 Hz. At the first level, writing activation maps reporting the contrast between copying characters and drawing symbols were generated for high‐frequency and low‐frequency characters for each participant. The activation maps were then entered into a random‐effect ANOVA to examine the effects of group and condition, and their interaction. To identify within‐group activation specific to handwriting, separate brain activation maps for males and females were computed separately, using one‐sample *t* tests computed during the ANOVA procedure. For the within‐group activation, the voxel‐wise threshold for statistically significant activation was set at q < 0.05, false discovery rate corrected for multiple comparisons with a minimum cluster extent of 20 contiguous voxels. For the between‐group comparison, the voxel wise threshold was set at *p* < .001, and *p* < .05 family‐wise error (FWE) corrected at the cluster level. Brain regions were estimated from the Talairach atlas (Talairach & Tournoux, 1988).

Brain‐behavior correlation analysis was conducted to further confirm that the sex differences in brain activity are linked to real handwriting practice in daily life. Pearson's correlation coefficients were calculated between brain activation and post‐imaging pen‐and‐paper writing performance. To do this analysis, contrast estimates (linear combination of *β* estimates) were extracted from functional ROIs showing sex differences in activation for each participant, which were then correlated with the writing completion time in high‐frequency and low‐frequency conditions. Because the correlation analysis was done twice (high‐frequency and low‐frequency conditions), the significance threshold was set at *p* < .025, corresponding to *p* < .05 after applying the Bonferroni correction for multiple comparisons.

#### 3.2.3 Lateralization analysis

Cerebral lateralization of the brain is a key hypothesis for sex differences in language (Clements et al., 2006; Gur et al., 2000; Hirnstein et al., 2013). Thus, a lateralization index (LI) analysis was also conducted using the LI toolbox (Wilke & Lidzba, 2007). Regions classically recognized to be associated with handwriting were selected as ROIs (Planton et al., 2017), including the intraparietal sulcus/superior parietal lobule (IPS/SPL, Left: −32, −38, 56; right: 32, −38, 56 in MNI coordinates), Exner's area (left: −22, −8, 54; right: 26, 0, 54), visual word‐form area (VWFA, left: −46, −62, −12; right: 46, −62, −12) and cerebellum (left: −4, −66, −16; right: 4, –66, −16). All the regions were created as spherical ROIs with a radius of 10 mm. To avoid the bias of a fixed threshold, a bootstrap method implemented in the LI‐toolbox was used, which allows calculation of the asymmetry index (AI) via thousands of comparisons across thresholds between the two hemispheres. The AI was defined by (Left−Right)/(Left +Right) for high‐frequency and low‐frequency characters, respectively. The weighted mean of AI values was first acquired for males and females. Following the method of previous studies (Gaillard et al., 2011; Xu, Yang, Siok, & Tan, 2015), the lateralization criteria were set as left hemisphere dominance: AI ≥0.2, right hemisphere dominance: AI ≤ −0.2, and bilateral representation: −0.2 < AI <0.2. Then, AI values were entered into a 2 (sex: male vs. female) by 2 (stimuli type: high‐frequency vs. low‐frequency) ANOVA to quantify the sex difference in brain lateralization. The statistical threshold for between‐group differences was set at *p* < .0125, corresponding to *p* < .05 for 4 ROIs after applying the Bonferroni correction for multiple comparisons.

#### 3.2.4 Functional connectivity analysis

Additional analyses were used to investigate how the interactions among writing‐related regions were modulated by the sex difference. A generalized psychophysiological interaction (gPPI) analysis (McLaren, Ries, Xu, & Johnson, 2012) was applied to compute the functional connectivity associated with sex differences. The gPPI analysis illustrates task‐dependent interaction between a seed region defined a priori and all voxels in the rest of the brain, based on multiple regression models (Friston et al., 1997). Functional regions of interest (ROIs) were defined based on the results showing sex differences in the brain activation analysis. For each ROI, a regression model was built using three task regressors (high‐frequency characters; low‐frequency characters and drawing symbols), three gPPI regressors for each condition, and a regressor representing the seed time series. At the first level, the gPPI parameter maps for high‐frequency and low‐frequency writing for each ROI were generated, and these were put into a 2 (sex: male vs. female) by 2 (stimuli type: high‐frequency vs. low‐frequency) ANOVA to examine sex differences. The threshold was set at *p* < .001 uncorrected at the voxelwise level and *p* < .05 with FWE correction at the cluster level.

Pearson's correlation coefficients between functional connectivity and post‐imaging behavioral scores were also computed. Functional parameters (gPPI estimates) were extracted from functional ROIs showing sex differences in functional connectivity for each participant, which were then correlated with the pen‐and‐paper writing time.

## 4 RESULTS

### 4.1 Behavioral results

#### 4.1.1 fMRI task performance

**Figure 1:**
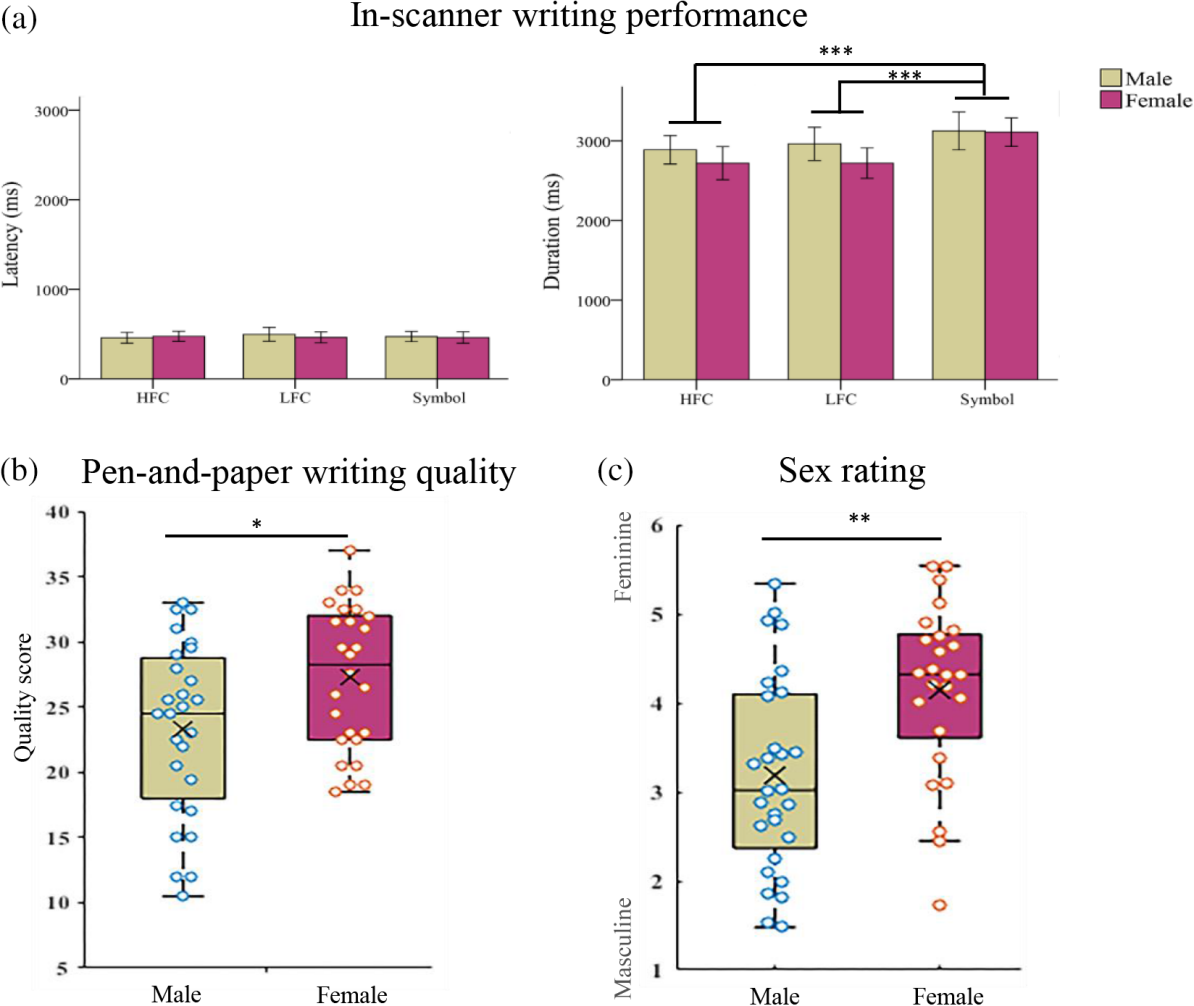
Behavioral results. Writing latency and duration during fMRI scan (a). Pen‐and‐paper test writing qualit**y** (b). Sex rating of the written script (c). HFC, high‐frequency characters; LFC, low‐frequency characters; ms, millisecond. Error bars denote the *SE* of the mean. Significant differences: * = *p* < .05; ** = *p* < .01; *** = *p* < .001

Figure 1 shows the behavioral results obtained for both groups during fMRI and post‐imaging. The reported fMRI results were based on 51 participants (Figure 1a), after screening for fMRI data quality issues as described in the Preprocessing section above. For writing latency during fMRI, neither the main effects of stimulus type (F [2,48] = 0.48, *p* = .619) and sex (F[1,49] = 0.06, *p* = .81), nor the stimulus type by sex interaction (F[2,48] = 1.43, *p* = .25) were significant. For writing duration, the main effect of stimulus type was significant (F[2,48] = 14.95, *p* < .001), but the main effect of sex (F[1,49] = 1.25, *p* = .27) and sex‐by‐stimulus type interaction (F[2,48] = 2.69, *p* = .078) were not significant. Post hoc pairwise comparisons showed that symbol drawing duration was significantly longer than writing high‐frequency characters (difference = 313 ms, *p* < .001) and writing low‐frequency characters (difference = 276 ms, *p* < .001), but there was no significant difference in duration between the two‐character conditions (difference = 37 ms, *p* = .161).

#### 4.1.2 Post‐imaging behavioral performance

The mean writing quality score (and *SD*) was 23.33 (6.66) for males and was 27.3 (5.5) for females, respectively, and females performed better than males (t[51] = 2.36, *p* = .022) (Figure 1b). The mean completion time (and *SD*) of high‐frequency characters was 53.09 ms (7.82 ms) for males and was 49.86 ms (8.98 ms) for females, while for low‐frequency characters, the time was 52.92 ms (9.48 ms) for males and was 48.98 ms (9.83 ms) for females. However, there were no significant sex differences for the writing time (high‐frequency: t(51) = 1.4, *p* = .168; low‐frequency: [t(51) = 1.48, *p* = .144]).

In addition, the average accuracy (and *SD*) of the dichotomous sex judgment was 0.62 (0.07), which was significantly higher than the chance level (t[44] = 11.99, *p* < .001). For the masculinity/femininity evaluation of written script, the average score (and *SD*) was 3.35 (1.14) for males, and 4.35 (1.14) for females, respectively, with the female writing rated significantly more feminine (t(51) = 3.16, *p* = .002) (Figure 1c).

#### 4.1.3 Whole‐brain activation analysis

**Figure 2:**
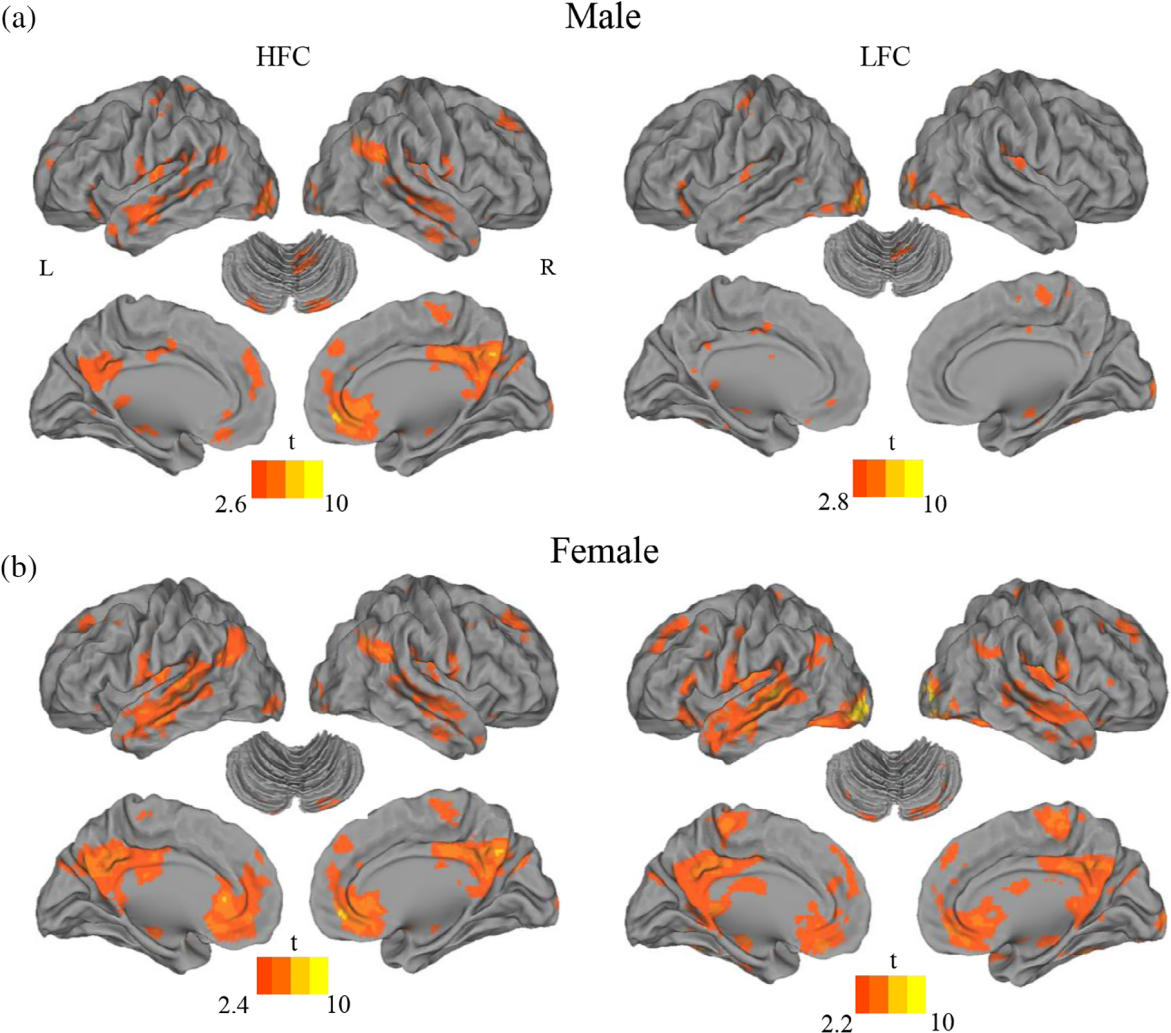
Brain regions associated with copying of Chinese characters in males (a) and in females (b). L, left; R, right; HFC, high‐frequency characters; LFC, low‐frequency characters

First, one‐sample *t* tests revealed within‐group activation maps of handwriting, showing that when contrasted with drawing symbols, writing Chinese characters elicited a similar and widespread activation pattern in both males and females, involving the precentral gyrus, superior/middle/inferior frontal gyrus, postcentral gyrus, superior and middle temporal gyrus, fusiform gyrus, and the cerebellum (Figure 2). These regions were in broad agreement with previous findings of the neural correlates of handwriting (Planton et al., 2013; Planton et al., 2017; Purcell et al., 2011).

**Figure 3:**
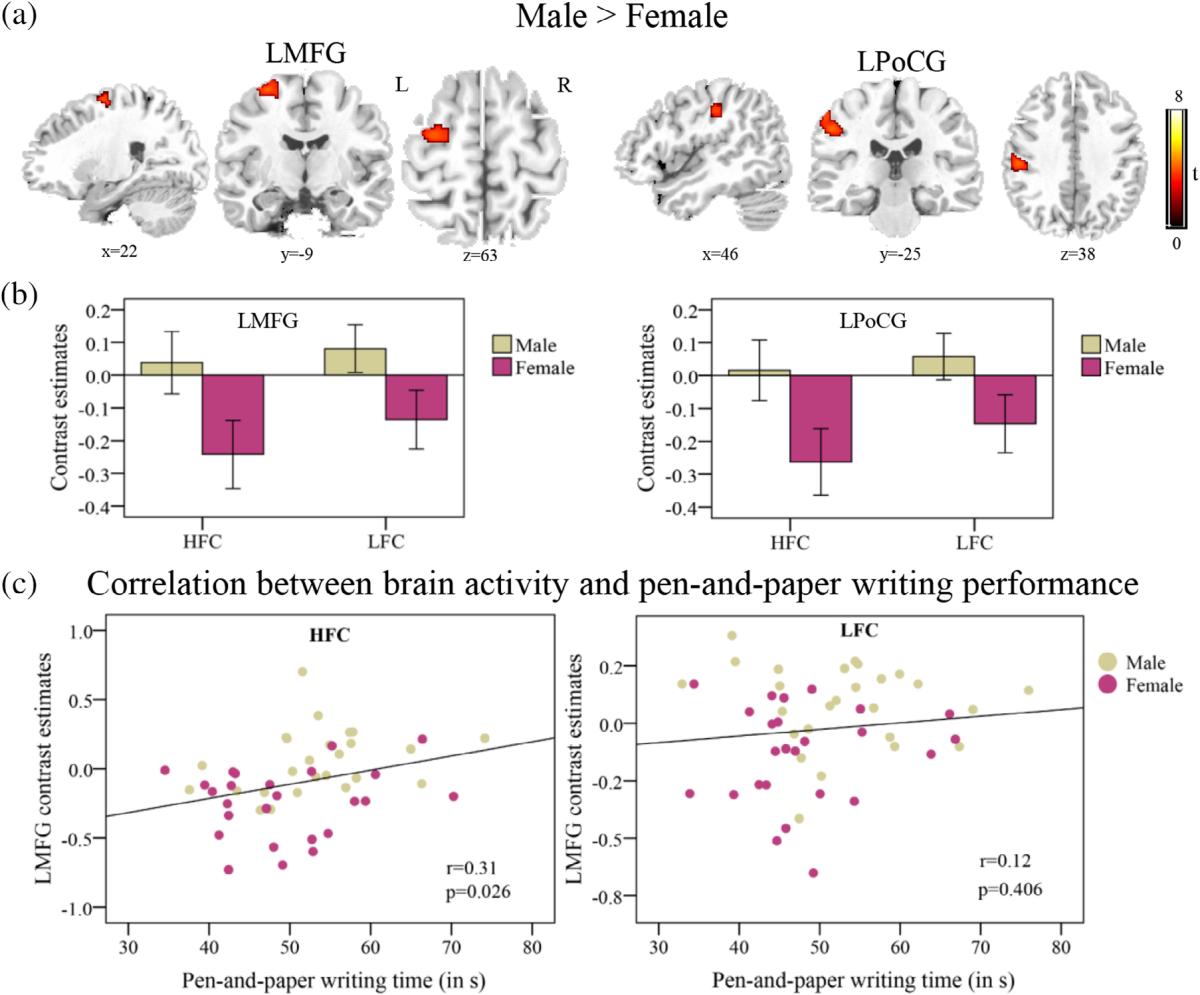
Sex differences in brain activation during handwriting. Brain regions showing group difference (a). Mean contrast estimates for the two groups extracted from the regions showing sex differences (b). Scatter plots for the correlation between BLOD responses of the LMFG and the pen‐and‐paper writing time (c). LMFG, left middle frontal gyrus, LPoCG, left postcentral gyrus. HFC, high‐frequency characters; LFC, low‐frequency characters; L, left; R, right; s, second

Then, the between‐group comparison revealed that males showed greater brain activation in the posterior left middle frontal gyrus (MFG) (Talairach: −28, −5, 59, BA 6), corresponding to Exner's area, and the left postcentral gyrus (PoCG) (Talairach: −52, −24, 38, BA2) (Figure 3a). No significantly greater brain activation was detected in females compared to males, and the sex by stimulus type interaction was not significant. The mean BLOD responses of the left MFG and left PoCG were presented in Figure 3b.

To verify that the differences in activation were specific to handwriting, activation in these two regions was also investigated in the symbol drawing condition. Independent two‐sample *t* tests indicated that no significant sex differences were evident in the activation of Exner's area (t[49] = 0.42, *p* = .675) and the left PoCG (t[49] = 1.12, *p* = .268).

Correlation analysis revealed that greater activity in Exner's area was marginally significantly correlated with slower writing completion time during pen‐and‐paper writing in the high‐frequency condition (r = 0.31, *p* = .026), but not in the low‐frequency condition (r = 0.12, *p* = .406) (Figure 3c). However, the activity in the left PoCG was not significantly correlated with the pen‐and‐paper writing completion time in the high‐frequency condition (r = 0.27, *p* = .053) and the low‐frequency condition (r = 0.21, *p* = .14).

#### 4.1.4 Lateralization analysis

**Figure 4:**
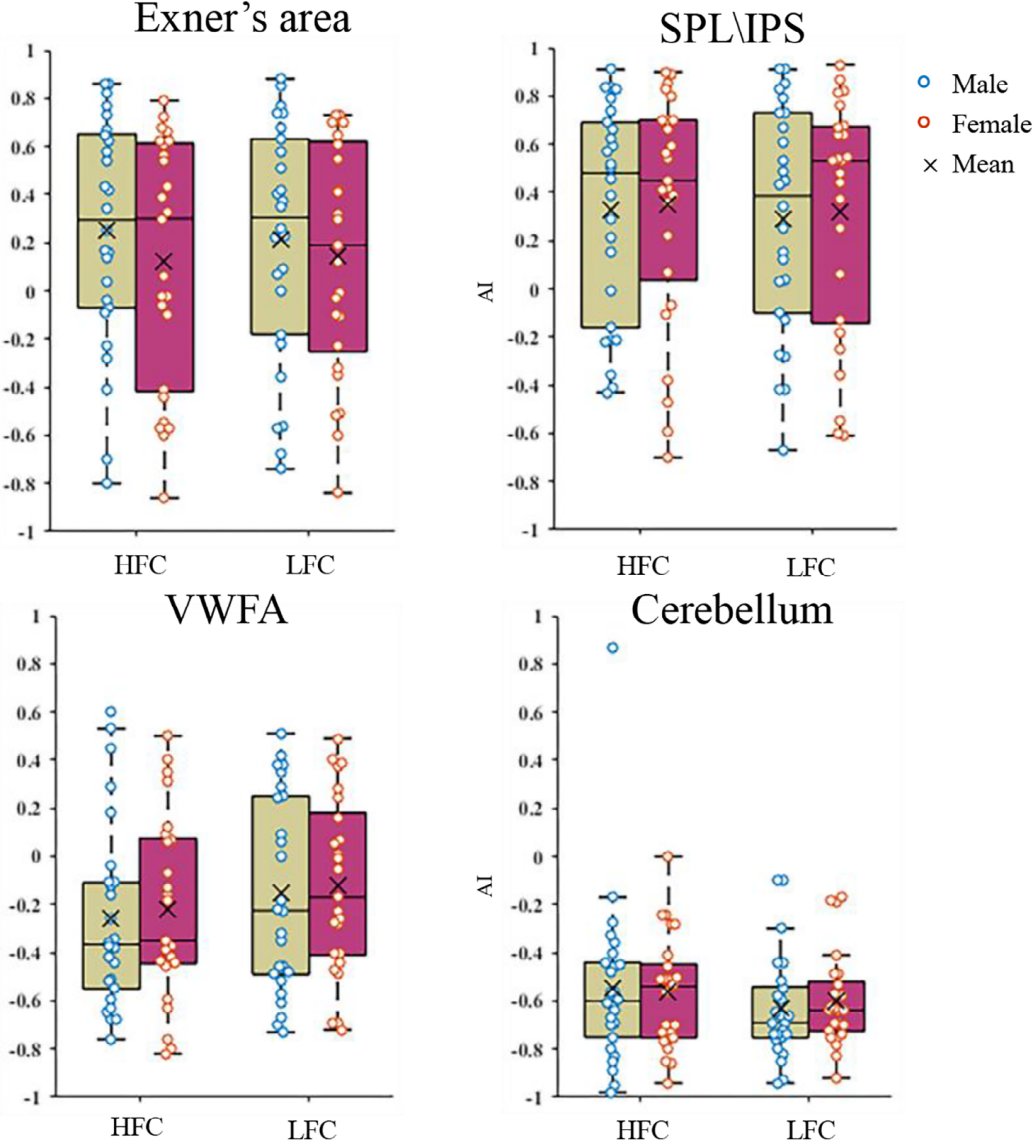
Asymmetry index (AI) values for each ROI investigated and for each participant. SPL\IPS, superior parietal lobule\inferior parietal sulcus; VWFA, visual word form area; HFC, high‐frequency characters; LFC, low‐frequency characters

Figure 4 shows the AI value in each ROI for each participant. Males and females exhibited a comparable asymmetrical pattern in all ROIs except within Exner's area. The activity in Exner's area was left‐lateralized in males (mean AI = 0.25 and 0.21 for high‐frenquency and low‐frequency, respectively), but was bilateral in females (AI = 0.12 and 0.14). The activity in the superior parietal lobule was left‐lateralization in males (high‐frequency: AI = 0.33 and low‐frequency: AI = 0.29) and in females (high‐frequency: AI = 0.34 and low‐frequency AI = 0.31). The activity in *VWFA* was right‐lateralized in the high‐frequency condition in males (AI = −0.25) and in females (AI = −0.22), but was bilateral in the low‐frequency condition in males (AI = −0.15) and in females (AI = −0.12). The activity in the cerebellum was right‐lateralized in both males (high‐frequency: AI = −0.55 and low‐frequency AI = −0.63) and females (high‐frequency AI = −0.57 and low‐frequency AI = −0.6). However, no significant gender differences in AI values were observed for any of the ROIs investigated.

#### 4.1.5 Functional connectivity analysis

**Figure 5:**
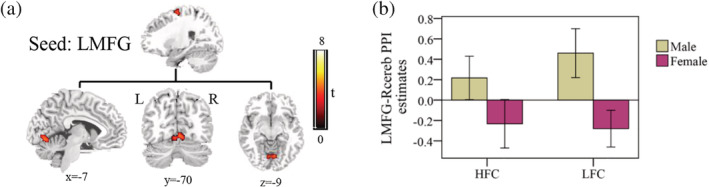
Sex differences in functional connectivity. Sagittal, coronal, and axial views of the regions showing sex differences based on connectivity analysis involving the left middle frontal gyrus as the seed region (a). The gPPI parameter estimates of connectivity between the left middle frontal gyrus and right cerebellum (b). HFC, high‐frequency characters; LFC, low‐frequency characters; LMFG, left middle frontal gyrus

As only Exner's area showed reliable sex differences across different writing status, it was selected as the seed for functional connectivity analysis. The result revealed that males had stronger functional connectivity between Exner's area and the right cerebellum (peak at 4 –72 −8) (Figure 5a). No other group differences were detected. Post hoc examination showed that males had positive connectivity, while females had negative connectivity (Figure 5b). However, correlation analysis revealed that functional connectivity between Exner's area and the right cerebellum was not correlated with pen‐and‐paper writing completion time in the high‐frequency condition (r = 0.08, *p* = .56) and the low‐frequency condition (r = 0.14, *p* = .34).

## 5 DISCUSSION

The aim of this study was to explore the neural basis underlying sex differences in handwriting, a hallmark of human language and motor skill. Using fMRI in a copying task, this study compared brain activation and functional connectivity during handwriting between males and females whose age, education level, reading, cognitive and visual‐motor skills were well‐matched. In line with previous findings in other language systems (Reynolds et al., 2015; Scheiber et al., 2015), behavioral results showed significant sex differences of handwriting outputs *in Chinese*. Functional MRI measures revealed differences in the activation of the left posterior middle frontal gyrus (Exner's area), irrespective of language material. Moreover, functional connectivity between Exner's area and the cerebellum was different between males and females, suggesting the sex difference in neural integration underlying handwriting. The activity in Exner's area was also found to be correlated with actual writing behaviors, suggesting that Exner's area might be a neural substrate of sex differences in handwriting. Collectively, this study unfolds a novel neural signature of sex differences in a hallmark of human behavior, highlighting the role of sex in handwriting in scientific research and clinical application.

The study was carefully designed to acquire multiple behavioral measures for quantifying sex differences in the actual written outputs. Consistent with previous studies (Beech & Mackintosh, 2005), both the dichotomous judgment and the extent judgment using a 7‐point scale differentiate the written script of females from that of males. Furthermore, females had higher scores than males in writing quality, which agrees with previous findings (Kaufman, Kaufman, Liu, & Johnson, 2009; Reynolds et al., 2015). Overall, the behavioral measures confirmed that participants in the current study were categorically different in their written products and thus presumably the underlying neural processes.

The fMRI measures showed hyperactivity in Exner's area in males compared to females during handwriting. Put another way, females had less activation in this area when writing character relative to the symbol‐drawing baseline, whereas a similar reduction was not apparent in males. The behavioral performance during fMRI was comparable between males and females, excluding the possibility that this activation difference was derived from task performance, rather than from the sex difference itself. This difference was independent of writing materials (high‐frequency vs. low‐frequency characters). Moreover, the significant correlations between the activity in Exner's area and behavioral performance metrics reinforced the relevance of the brain activation in this area to writing, implying that Exner's area is a specific neural substrate of sex differences in handwriting.

Evidence from pure agraphia (Anderson, Damasio, & Damasio, 1990; Exner, 1881; F. E. Roux et al., 2009) and cortical electrical stimulation (F. E. Roux et al., 2009) has indicated that the posterior part of the left middle frontal gyrus (Exner's area) is a writing‐specific region, despite the challenge of its anatomical localization (Matsuo et al., 2003). Neuroimaging studies in healthy subjects have also demonstrated activation in this region after controlling for language and low‐level motor processing in various writing tasks (Planton et al., 2013; Planton et al., 2017; Purcell et al., 2011). Furthermore, a recent fMRI study demonstrated that Exner's area was the only region that showed writing‐specific lateralization (Planton et al., 2017). Therefore, it is reasonable to observe reduced activation in this region during handwriting compared to drawing, because handwriting of characters is a highly practiced behavior relative to drawing symbols. Functionally, Exner's area is thought to serve the connection between orthographic code and motor programs during handwriting (F. E. Roux et al., 2009). It has been found to represent motor gestures of written scripts that universally exist in alphabetic languages and Chinese (Nakamura et al., 2012). In this sense, relatively higher activation in Exner's area in males might be associated with lower quality of the representation of motor gestures for written scripts that disturbs the transformation from orthographic to motoric programs during handwriting.

Alternatively, several fMRI studies have demonstrated that the activity in the middle frontal sulcus was modulated by word length in alphabetic languages (Rapp & Dufor, 2011; Rapp & Lipka, 2011) and by the number of strokes in Chinese characters (Chen et al., 2007), suggesting that this area houses orthographic working memory. However, Planton et al. (2017) reported that Exner's area was not activated in an oral spelling task which necessarily requires orthographic working memory processing. Similarly, the dynamic presentation of visual words produced a significant priming effect in Exner's area, but the static presentation did not (Nakamura et al., 2012). Consequently, the role of Exner's area in handwriting might be close to the motor output buffer, rather than the temporary storage of pure orthographic information. These findings also support the view that sex differences in handwriting might originate from the interface between orthographic and motor processes, but not from pure orthographic storage.

Brain lateralization provides a key biological account for sex differences in language processing. In the present study, lateralization analysis demonstrated that males exhibited left‐lateralized activation in Exner's area during handwriting, while females exhibited bilateral. This pattern is consistent with previous findings of sex differences in other language tasks (Baxter et al., 2003; Burman et al., 2013; S. E. Shaywitz et al., 1998). In particular, a recent study has reported left‐lateralized activation in Exner's area during handwriting (Planton et al., 2017). Our results extend the findings of the functional lateralization of Exner's area, highlighting the need to take sex differences into account when the role of brain lateralization in handwriting is tested. However, it should be noted that the direct comparison of asymmetry indices failed to show significant sex differences in any ROIs. One possibility is that sex differences in functional asymmetry during language processing is small (Hirnstein et al., 2013), and the statistical power of this study might not be strong enough to detect the subtle effect due to the small sample size. Thus, further studies with large a sample size are needed to quantify the magnitude of sex differences in brain lateralization during handwriting. Unexpectedly, both males and females exhibited *right‐lateralized* or bilateral activation in VWFA, contrary to the fMRI study by Planton et al. (2017) who reported a left‐lateralized activation. The difference in written material might account for this discrepancy to some extent. The Chinese characters used by this study are more visually complex stimuli than the French words used by Planton et al. (2017), which would lead to a difference in demand for visual–spatial processing. The visual representation of Chinese characters is more likely to be bilateral in Chinese written systems (Wu, Ho, & Chen, 2012), and thus the visual word regions show bilateral activation during Chinese writing (Cao & Perfetti, 2016; Yang et al., 2018; Yang et al., 2019). This claim warrants testing in further studies comparing brain activation patterns between Chinese and alphabetic writing systems.

The study also investigated how and to what extent functional interactions between writing‐related regions contribute to sex differences in handwriting. The gPPI analysis indicated that the connectivity between Exner's area and the right cerebellum was stronger in males than in females. Specifically, males showed positive connectivity during the writing task compared to the symbol‐drawing baseline, whereas females showed a negative pattern. Cerebellum is a key neural substrate of handwriting that has been reported in multiple writing tasks, and it has been proposed that it supports the motor execution in handwriting (Planton et al., 2013; Planton et al., 2017). As a result, the connectivity between Exner's area and the cerebellum would represent the neural circuit supporting the communication between motor planning to execution. However, we found that the connectivity between Exner's area and the cerebellum was not correlated with real pen‐and‐paper writing performance. We suspect that the connectivity difference might only reflect different writing strategies, not processing efficiency. In other words, males and females might adopt different neural pathways to achieve a similar level of behavioral performance. This hypothesis needs to be tested in future studies.

Another finding of this study is that males showed increased brain activity in the left postcentral gyrus compared to females. Like the pattern of Exner's area, this difference originated from lower activation in writing character relative to drawing symbols in females. Although the left PoCG has been consistently found to be involved in handwriting (Katanoda et al., 2001; Planton et al., 2013; Purcell et al., 2011), its function remains unclear. One suggestion is that the left postcentral gyrus is engaged as part of the somatosensory feedback, a vital component of handwriting (Sakurai, et al., 2007). For example, writing pressure is known to vary throughout a specific handwriting task, typically increasing progressively toward a maximum shortly before task completion (Kao, 1983). Further studies are needed to confirm whether and why there is a sex difference in the postcentral gyrus during handwriting.

Finally, our findings have methodological implications for neuroscience research of handwriting, supporting the view that sex differences should be taken into account in neuroscience research studies (Bale & Epperson, 2017; McCarthy, Woolley, & Arnold, 2017). For example, some handwriting‐related disorders have been found to show strong male prevalence, such as dysgraphia (Karlsdottir & Stefansson, 2002) and dyslexia (Arnett et al., 2017). The results from group comparisons in language and motor experiments might be biased by sex differences if the number of male and female participants is not controlled. Thus, it is best to account explicitly for the factor of sex in the design of neuroimaging studies of handwriting.

## 6 LIMITATIONS

The sample size was relatively small for a sex differences study, which would increase the risk of false‐positive detection (Etchell et al., 2018; Kaiser, Kuenzli, Zappatore, & Nitsch, 2007). Although behavioral studies have consistently reported sex differences in handwriting across age and historical time (Reilly et al., 2019; Reynolds et al., 2015), whether, and to what extent the neural basis of sex differences exists among the population needs to be confirmed in further studies with larger sample sizes.

Secondly, development is a critical factor influencing the pattern of sex differences at both behavioral (Vlachos & Bonoti, 2006; Zell et al., 2015) and neural (Burman et al., 2013) levels. This study only recruited a group of adult participants with a narrow range of age, and therefore it is unclear whether the neural difference in handwriting found by the present study could apply to people of different ages.

Thirdly, we only measured the motor response duration at the whole character level, and thus it is unclear how and to what extent sex differences occur at the sub‐character level. According to the homothety principle, the motor time of individual letters would be invariant, although the duration of the whole word may vary (Viviani & Terzuolo, 1982). A prior study has demonstrated that dyslexics who have motor dysfunction of handwriting showed more variation in the time to write the individual letters of a word, failing to conform to the homothety principle (Pagliarini et al., 2015). It would be interesting to examine sex differences at the sub‐word level (e.g., radical or strokes in Chinese character) in light of the homothety principle, which may provide further insight into the source of sex differences in handwriting.

## 7 CONCLUSION

This study addressed the question of the neural basis of sex differences in handwriting, a unique form of language and motor behavior. Males and females were shown to have different utilization of a writing‐specific brain region, namely Exner's area, and this has a plausible link to behavioral sex differences in handwriting. The work has methodological implications for neuroimaging studies of handwriting in normal and patient participants.

## CONFLICT OF INTERESTS

The authors declare that they have no conflict of interest.

## AUTHOR CONTRIBUTIONS

Y.Y., Z.T.Z., F.T., S.J.G., and H.Y.B. conceived and designed the experiment. Y.Y., J.J.L., C.Y.G., and Z.T.Z. performed the experiment. Y.Y., Z.T.Z., N.Z.W., J.J.L., and R.T. performed the data analyses. Y.Y., Z.T.Z., and H.Y.B. co‐wrote the paper. Y.Y., Z.T.Z, F.T., S.J.G., G.C.S., N.Z.W., R.T., J.J.L., and H.Y.B. discussed the data and commented on the manuscript.

## Data Availability

The data that support the findings of this study are available from the corresponding author upon reasonable request.
